# Repression of the miR-627-5p by histone deacetylase 3 contributes to hypoxia-induced hepatocellular carcinoma progression

**DOI:** 10.7150/jca.58697

**Published:** 2021-07-03

**Authors:** Jun Wang, Runkun Liu, Yufeng Wang, Huanye Mo, Yongshen Niu, Qiuran Xu, Qingguang Liu

**Affiliations:** 1 Department of Emergency, The First Affiliated Hospital of Xi'an Jiaotong University, Xi'an 710061, China.; 2 Department of Hepatobiliary Surgery, The First Affiliated Hospital of Xi'an Jiaotong University, Xi'an 710061, China.; 3 The Key Laboratory of Tumor Molecular Diagnosis and Individualized Medicine of Zhejiang Province, Zhejiang Provincial People's Hospital, Affiliated People's Hospital, Hangzhou Medical College, Hangzhou 310014, China.

**Keywords:** Hepatocellular carcinoma, Hypoxia, HDAC3, miR-627-5p, Histone deacetylation

## Abstract

Hepatocellular carcinoma (HCC) is one of the most common solid tumors globally. Our previous studies revealed that miR-627-5p suppresses HCC progression via targeting BCL3/CCND1 pathway. However, the molecular mechanism by which miR-627-5p was downregulated in HCC remains to be further elucidated. As a hallmark of solid tumors, hypoxia results in the rapid growth, strongly potential invasion and high frequent metastasis of cancer cells. Hypoxia-inducible factors (HIFs), mainly including HIF-1 and HIF-2, are the classical transcription factors which mediate hypoxia-related gene transcription. Here, we demonstrated that miR-627-5p was repressed by hypoxia in a HIF-1-dependent manner in HCC cells. But HIF-1 regulated miR-627-5p expression not directly through the hypoxia-response element (HRE) sites of MIR627 gene. In contrast, histone deacetylase 3 (HDAC3) was identified as a HIF-1 target gene, and the occupancy of HIF-1 to HRE site was essential for hypoxia-mediated HDAC3 induction. And upregulated HDAC3 was closely related to the malignant clinical and pathological characteristics and worse prognosis of HCC. Furthermore, HDAC3-mediated histone deacetylation in promoter region of MIR627 was critical for hypoxia-mediated miR-627-5p repression. And miR-627-5p mediated the effects of hypoxic condition on HCC progression. Thus, this study has revealed that miR-627-5p was repressed by hypoxia under the mediation of HDAC3 in HCC, and there existed a HIF-1α/HDAC3/miR-627-5p/BCL3/CCND1 signal pathway in HCC.

## Introduction

Hepatocellular carcinoma (HCC), the most common primary liver cancer, has the characteristic of high morbidity and mortality 1, 2. It usually happens to people with hepatitis B or C, viral hepatitis combined with cirrhosis and liver cancer family history 3. Currently, the etiology and exact molecular mechanisms of HCC are not fully understood. Therefore, it is urgent to discover some new and practical therapeutic strategies for HCC.

Hypoxia is a characteristic feature of the tumor microenvironment 4. Intratumoral hypoxia is a driving force for HCC progression that is mediated by hypoxia-inducible factors (HIFs), which are heterodimeric proteins consisting of an O2-regulated HIF-1α, HIF-2α, or HIF-3α subunit and a constitutively expressed HIF-1β subunit 4-6. Increased HIF-1α expression in primary tumor biopsies is associated with decreased disease-free, and overall survival of HCC patients 7. In response to hypoxia, HIFs activate transcription of hundreds of genes that play key roles in angiogenesis, metabolic reprogramming, extracellular matrix remodeling, invasion and metastasis 8-11. HIFs bind to hypoxia response elements (HREs) containing the core sequence 5'-RCGTG-3' (R = A or G) in target genes, and recruit mediator subunits, histone modifying enzymes and chromatin remodeling complexes 12-14. HIFs-HRE binding-mediated gene transcription under hypoxia is a classical pattern for most of the hypoxia-induced or hypoxia-repressed genes 6.

Epigenetics is the study of any potentially stable and ideally heritable changes in gene expression or cellular phenotype without alterations in DNA sequences 15. Epigenetic modulations, including histone modification and RNA-based mechanisms, have been identified to regulate cancer progression 15. Histone deacetylases (HDACs) and microRNAs (miRNA) have emerged as two important epigenetic mediators in cancer cells 16, 17. The acetylation of histones is conducive to the dissociation of DNA and histone octamers, and the nucleosome structure is relaxed, so that various transcription factors and synergistic transcription factors can specifically bind with DNA binding sites and activate gene transcription, whereas deacetylation of histones has the opposite effects 18. Histone deacetylases (HDACs) were shown to associate with transcribed regions of active genes to prevent gene transcription 16. HDACs are separated into four groups based on their structures and functions, and HDAC3 is a member of class I 16, 19. HDAC3 is an enzyme responsible for the deacetylation of lysine residues on the N-terminal part of the core histones 20. HDAC3 has been identified as a mediator in transcriptional regulation of gene 19. In our previous studies, miR-627-5p has been identified as a tumor suppressor in HCC 21. And we demonstrate that B-Cell leukemia/lymphoma 3 (BCL3) is the downstream target of miR-627-5p 21. MiR-627-5p inhibits HCC progression through targeting BCL3/CCND1 pathway 21, 22. Nevertheless, the molecular mechanism by which miR-627-5p was downregulated in HCC remains unclear.

This study demonstrates that miR-627-5p is repressed by hypoxia in a HIF-1-dependent manner in HCC cells, but not directly through the HRE sites of MIR627 gene. Hypoxia induces HDAC3 expression through the binding of HIF-1 and HRE in HCC cells. Additionally, upregulated HDAC3 is closely related to the malignant clinical and pathological characteristics and worse prognosis of HCC. Furthermore, HDAC3-mediated histone deacetylation in promoter region of MIR627 is critical for hypoxia-mediated miR-627-5p repression. And overexpressed miR-627-5p reverses the promotion effects of hypoxic condition on HCC progression. Thus, this study has revealed a novel hypoxia-related mechanism in which hypoxia regulates HCC progression through HIF-1α/HDAC3/miR-627-5p/BCL3/CCND1 signal.

## Materials and methods

### Tissue samples

Ninety pairs of HCC tissue samples and corresponding adjacent non-tumor tissue samples, which were histopathologically confirmed, were randomly collected from patients who underwent surgery in the First Affiliated Hospital of Xi'an Jiaotong University. All of the patients did not receive any preoperative treatment, such as chemotherapy, radiotherapy, and radiofrequency ablation. All of the samples were stored at -80 °C. Our study got approval from the Ethics Committees of the First Affiliated Hospital of Xi'an Jiaotong University and written informed consent was obtained from all patients.

### Cell culture

Human HCC cells (HepG2, Hep3B, Huh7 and SMMC-7721) and the normal hepatic cell line LO2 were maintained in an incubator (37 °C, 5% CO_2_) and cultured in DMEM (Gibco, Grand Island, NY, USA) supplemented with 10% FBS (Gibco, Grand Island, NY, USA) and 1% penicillin-streptomycin (Invitrogen, CA, USA).

### Cell transfection

The pLKO.1-puro vectors encoding a non-targeting control (NTC) short hairpin RNA (shRNA) or shRNA targeting either HIF-1α (shHIF-1α), HIF-2α (shHIF-2α) or HDAC3 (shHDAC3) were purchased from Sigma-Aldrich. Then lentivirals were constructed. The pcDNA3.1/HDAC3 vector (GenePharma, Shanghai, China) was applied to overexpress HDAC3. And the empty pcDNA3.1 vector was used for the control. The miR-627-5p mimics (#HmiR-SN0735) and miRNA scrambled control (# CmiR-SN0001-SN) were purchased from FulenGen (Guangzhou, China). All of the experimental operations were based on the product specifications.

### Reverse transcription and quantitative real-time PCR (RT-qPCR)

RNA was extracted using TRIzol (Invitrogen) and cDNA synthesis was performed according to the manufacturer's instructions (Applied Biosystems). RT-qPCR was performed using SYBR Green qPCR Master Mix (Bio-Rad). The expression of each target mRNA relative to 18S rRNA was calculated based on the threshold cycle (Ct) as 2^-Δ(ΔCt)^, where ΔCt = Ct_target_ - Ct_18S_ and Δ(ΔCt) = ΔCt_treatment_ - ΔCt_control_. PCR primers for miR-627-5p (MQPS0001996-1-200) were purchased from RiboBio. Nucleotide sequences of PCR primers for HIF-1α: Forward: 5'-CCACAGGACAGTACAGGATG-3', Reverse: 5'-TCAAGTCGTGCTGAATAATACC-3'. Nucleotide sequences of PCR primers for HIF-2α: Forward: 5'-TTGCTCTGAAAACGAGTCCGA-3', Reverse: 5'-GGTCACCACGGCAATGAAAC-3'. Nucleotide sequences of PCR primers for HDAC3: Forward: 5'-CCTGGCATTGACCCATAGCC-3', Reverse: 5'-CTCTTGGTGAAGCCTTGCATA-3'. Nucleotide sequences of PCR primers for 18S rRNA: Forward: 5'-CGGCGACGACCCATTCGAAC-3', Reverse: 5'-GAATCGAACCCTGATTCCCCGTC-3'. The primers were synthesized by Sangon Biotech (Shanghai).

### Chromatin immunoprecipitation (ChIP) assay

Hep3B and SMMC-7721 cells were incubated at 20% or 1% O_2_ for 16h, cross-linked in 3.7% formaldehyde for 15 minutes, quenched in 0.125 M glycine for 5 minutes, and lysed with SDS lysis buffer. Chromatin was sheared by sonication, and lysates were precleared with salmon perm DNA/protein A agarose slurry (Millipore) for 1 hour and incubated with antibodies against IgG (2 μg/sample, NBP2-36463, Novus), HIF-1α (2 μg/sample, NB100-479, Novus), HIF-1β (2 μg/sample, NB100-110, Novus), HIF-2α (2 μg/sample, NB100-122, Novus), H3K9ac (2 μg/sample, NB21-1017, Novus), H3 (2 μg/sample, NB500-171, Novus) or HDAC3 (2 μg/sample, NB100-1669, Novus) in the presence of protein A-agarose beads overnight. After serial washing of the agarose beads with low-salt, high-salt, and LiCl buffers, DNA was eluted in 1% SDS with 0.1M NaHCO3, and cross links were reversed by addition of 0.2M NaCl. DNA was purified by phenol-chloroform extraction and ethanol precipitation, and analyzed by qPCR. The primers were shown as below. MIR627-HRE-1: Forward: 5'-GGATTAATTCCCCCATTGCT-3', Reverse: 5'-GTACCCCCTCATCCATGTTG-3. MIR627-HRE-2: Forward: 5'-CTCCCAAAGTGCTGGGATTA-3', Reverse: 5'-CTTCCCTTCGTTGGGTAACA-3'. HDAC3-HRE: Forward: 5'-CTTCCCAAATGCCTTCACC-3', Reverse: 5'-ACCGGATAGACCAGTGGACA-3'. Primers used for MIR627 gene promoter PCR: Forward: 5'- TCCTGCCAGCAGTGTATGAG-3', Reverse: 5'-ACCAGGTATCCAACCTCAGC-3'.

### Transwell migration and invasion assays

After being transfected with plasmids for 48h, the cells were seed into Transwell chambers (8 mm pore size, Corning, USA) containing 200μl medium with 1% FBS. The lower chambers were added with 800μl medium containing 10% FBS. For detection of invasion ability, Transwell chambers were pre-coated with Matrigel. 24 h later, cells passed through the membrane were stained with crystal violet (0.1%) and counted.

### Cell proliferation assay

For CCK-8 assay, hepatocellular carcinoma cells (1 × 10^4^ cells/well) were seeded in 96-well plates and incubated with 10 μL of Cell Counting Kit-8 (CCK-8, Dojindo Laboratories, Dojindo) solution at indicated time points. Absorbance was detected at 450 nm using a microplate reader (Multiskan FC, Thermo Fisher Scientific). For EdU assay, Cell-Light™ EdU Apollo®567 In Vitro Imaging Kit (RiboBio Co., Ltd. Guangzhou, China) was used. Briefly, transfected HCC cells (1×10⁠^5^) were cultured in 96-well plates. Cells were incubated with EdU labeling medium at a moderate concentration for 2h. Then, the cells were fixed with 4% paraformaldehyde, glycine, and 0.5% TritonX-100 in PBS. Next, cells were stained with 100 μL Apollo dye solution for 30 min at room temperature. The cells were subsequently stained using Hoechst and incubated for 30 min. The photos were taken on a microscope. The percentage of EdU positive cells was calculated using ImageJ software.

### Luciferase reporter assay

The oligonucleotide spanning a potential HIF-1 binding site on MIR627 gene or HDAC3 gene promoter was inserted into plasmid pGL2-promoter. The oligonucleotides of wild HDAC3-HRE were mutated from GCGTG to GAAAG to construct the mutant type of HDAC3-HRE. The pGL2-promoter contained a basal SV40 promoter driving firefly luciferase expression. The control reporter plasmid pSV-Renilla encoded renilla luciferase expression. All of the plasmids were purchased from SwitchGear Genomics. Hep3B and SMMC-7721 cells were co-transfected with pGL2- promoter and pSV-Renilla. And 24h after transfection, the cells were exposed to 20% or 1% O_2_ for 24h. Luciferase activities were determined with a multiwell luminescence reader (PerkinElmer) using the Dual-Luciferase Reporter Assay System (Promega) and was presented as the ratio of firefly/Renilla luciferase activity.

### Western blot

Total proteins were isolated from cells with RIPA buffer (Beyotime, Hangzhou, China). The 10% SDS-PAGE gels separated protein, then transferred the protein to PVDF membranes (Millipore, Billerica, MA, USA). After being blocked by 5% nonfat milk for 2h, antibodies for HDAC3 (1:1000, ab137704, Abcam, USA), and HIF-1α (1:1000, ab228649, Abcam, USA), HIF-2α (1:1000, ab109616, Abcam, USA), BCL3 (1:1000, ab216877, Abcam, USA), CCND1 (1:1000, ab226977, Abcam, USA), β-actin (1:1000, sc-8432, Santa Cruz, USA) were used to incubate membranes at 4 °C overnight. Then, the membranes were incubated by the HRP-conjugated secondary antibodies (1:1000, Proteintech, USA) for 1-2h. The blots were detected using an enhanced chemiluminescence reagent (Millipore, Billerica, MA, USA).

### Statistical analysis

Graphpad Prism 8.0 (San Diago, CA, USA) and SPSS 20.0 (SPSS, Inc., Chicago, IL, USA) were applied to analyze the data. All of the data were presented as mean ± S.D. Statistical methods in this study included Student's *t* test, one-way ANOVA, Chi-square test, Kaplan-Meier method, log-rank test and Pearson's correlation coefficient analysis. The difference with* P <* 0.05 was considered to be statistically significant.

## Results

### MiR-627-5p is inhibited by hypoxia in HCC cells

**Figure 1:**
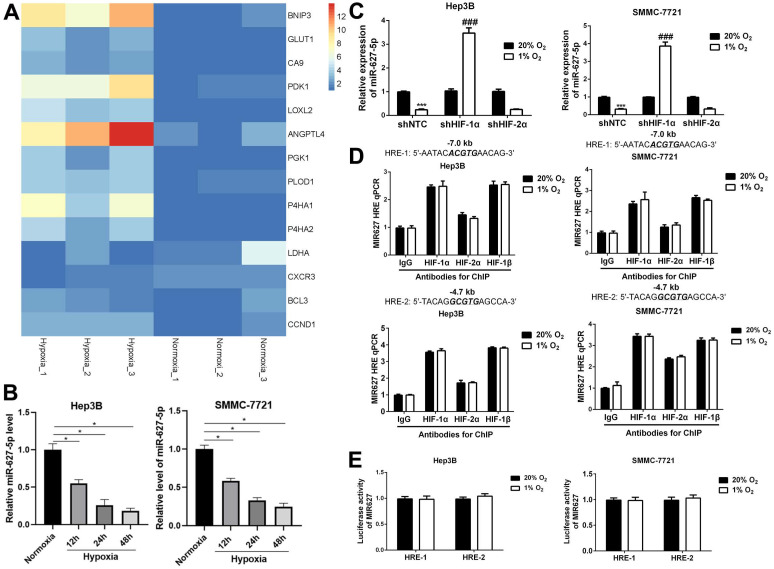
** MiR-627-5p is inhibited by hypoxia in HCC cells.** (**A**) Heat map for hypoxia-induced genes Hep3B cells. Chip Analysis on Gene Expression was conducted in Hep3B cells exposed to normoxia (20% O_2_) or hypoxia (1% O_2_) for 24h. BCL3 and CCND1 mRNA expression were induced by hypoxia. The samples were triplicated. (**B**) Hep3B and SMMC7721 cells were exposed to normoxia (20% O_2_) and hypoxia (1% O_2_) for 12h, 24h and 48h. And miR-627-5p expression was determined by RT-qPCR analysis (mean ± SD; *n* = 3). *P < 0.05 vs. Normoxia (two-way ANOVA) (**C**) The HIF-1α or HIF-2α knockdown subclones of Hep3B and SMMC-7721 were exposed to 20% or 1% O_2_ for 24 hours, followed by analysis of miR-627-5p levels by RT-qPCR (mean ± SD; *n* = 3). ***P < 0.001 vs. NTC at 20% O_2_; ###*P* < 0.001 vs. NTC at 1% O_2_ (two-way ANOVA). (**D**) Hep3B and SMMC-7721 cells were exposed to 20% or 1% O_2_ for 16h, and ChIP assays were performed using the indicated antibodies. Primers encompassing candidate HIF binding sites located 7.0 kb 5'and 4.7 kb 5' to the MIR627 gene transcription start site were used for qPCR and results were normalized to the first lane (mean ±SD; *n* = 3). There was no significant change (Student's *t* test). (**E**) The oligonucleotide spanning HRE-1 site or HRE-2 site was inserted into the reporter plasmid pGL2-promoter respectively, in which a basal SV40 promoter drives firefly luciferase expression. Hep3B and SMMC-7721 cells were co-transfected with pGL2/MIR627-HRE and exposed to 20% or 1% O_2_ for 24h. The ratio of firefly/Renilla luciferase activity was analyzed (mean ± SD; *n* = 3). There was no significant change (Student's *t* test).

Previously, our published data demonstrated that miR-627-5p is a tumor suppressor for HCC, and BCL3 is the downstream target of miR-627-5p in HCC, and miR-627-5p suppresses HCC progression by targeting BCL3/CCND1 signaling pathway 21, 22. Here, we attempted to explore the potential mechanism by which miR-627-5p was downregulated in HCC. Interestingly, our previous microarray data (GSE155505) 23 indicated that BCL3, CCND1 and some confirmed HIFs-target genes were induced by hypoxia in Hep3B cells (Figure 1A and Table 1). Given that BCL3 and CCND1 were negatively regulated by miR-627-5p in HCC, we hypothesized that miR-627-5p was inhibited by hypoxia. Hep3B and SMMC7721 cells were exposed to normoxia (20% O_2_) and hypoxia (1% O_2_) for 12h, 24h and 48h. And miR-627-5p expression was determined by RT-qPCR analysis. Data revealed that miR-627-5p was inhibited by hypoxia in a time-dependent manner (*P*<0.05, Figure 1B).

It's well known that HIF-1α/HIF-2α are key transcriptional factors under hypoxia. Then, we attempted to explore whether miR-627-5p was a HIF-1α/HIF-2α target gene. Hep3B and SMMC7721 cells were stably transfected with lentiviral vectors encoding a non-targeting control (NTC) short hairpin RNA (shRNA) or shRNA targeting either HIF-1α (shHIF-1α) or HIF-2α (shHIF-2α), then RT-qPCR analysis and Western blot were applied to verify the knockdown efficiency (*P*<0.05, Supplementary Figure 1). Subsequently, miR-627-5p expression was investigated in these subclones. RT-qPCR analysis indicated that miR-627-5p expression was increased under hypoxia when HIF-1α was silenced in Hep3B and SMMC-7721 cells, and there was no significant change in HIF-2α-knockdown subclone (*P*<0.05, Figure 1C). Furthermore, ChIP assay was applied to explore whether HIF-1α regulated miR-627-5p expression by directly targeting the HRE sites of MIR627 gene promoter region. Unfortunately, the data indicated that neither HIF-1α nor HIF-1β was able to directly target these HRE sites (Figure 1D). In addition, to further test whether HIF-1α regulated miR-627-5p through targeting HRE sites, the oligonucleotide spanning a HIF-1 binding site was inserted into the reporter plasmid pGL2-promoter, in which a basal SV40 promoter drives firefly luciferase expression. Hep3B and SMMC-7721 cells were co-transfected with pGL2/pri-miR-627-5p-HRE and pSV-Renilla, in which the basal SV40 promoter drives Renilla luciferase expression, and exposed to 20% or 1% O2 for 24h. The ratio of firefly/Renilla luciferase activity showed no significant change in both hypoxic Hep3B and SMMC-7721 cells (Figure 1E). Thus, we demonstrate that hypoxia regulates miR-627-5p in a HIF-1α-dependent manner in HCC cells, but HIFs are not able to directly bind to the MIR627 gene to regulate its transcription activity.

### Upregulated HDAC3 in HCC indicates poor prognosis

**Figure 2:**
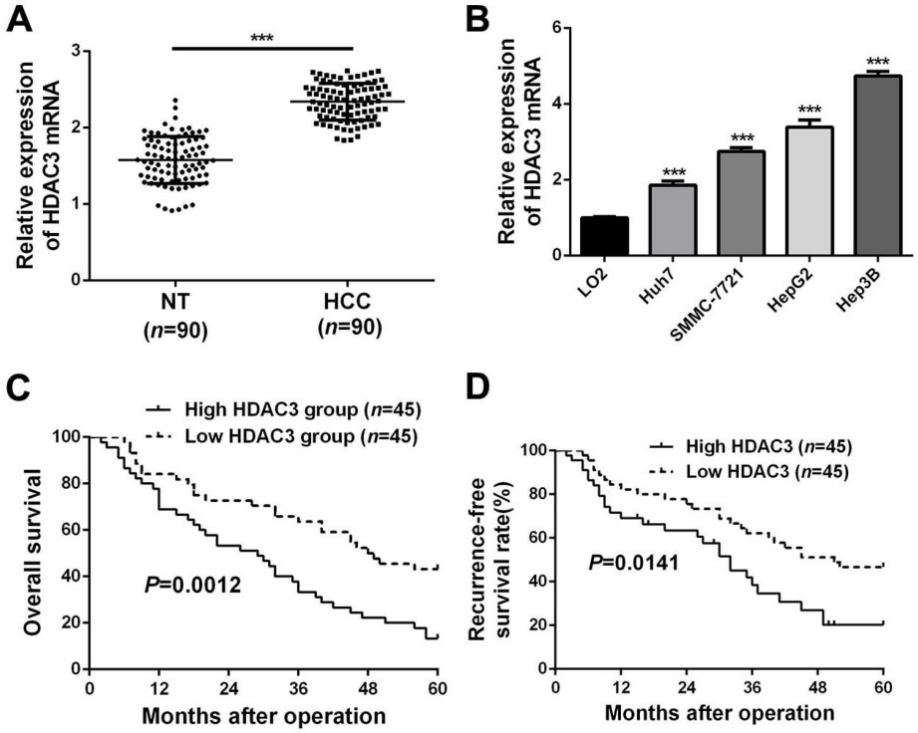
** Upregulated HDAC3 in HCC indicates poor prognosis.** (**A**) RT-qPCR analysis was conducted to explore the expression of HDAC3 in HCC tissues (*n*=90) and corresponding adjacent non-tumor (NT) tissues (*n*=90) (***P<0.001, Student's *t* test). (**B**) RT-qPCR analysis revealed that the expression of HDAC3 was notably increased in HCC cell lines (HuH7, SMMC-7721, HepG2 and Hep3B) compared to that in a normal hepatocyte cell line (LO2) (mean ± SD; *n* = 3) (***P<0.001, two-way ANOVA). HCC patients with higher HDAC3 expression had worse (**C**) 5-year overall survival and (**D**) recurrence-free survival than those with lower HDAC3 expression (Log-rank test).

RT-qPCR analysis data from HCC tissues and adjacent non-tumor tissues showed that HDAC3 is significantly upregulated in HCC tissues (*P*<0.05, Figure 2A and Supplementary Figure 2), which was consistent with the data from public dataset TCGA (*P*<0.05, Supplementary Figure 3A). In addition, HDAC3 was significantly upregulated in HCC cell lines, compared to the normal hepatic cell LO2 (*P*<0.05, Figure 2B). Clinically, the correlation analysis manifested that abnormally expressed HDAC3 was closely correlated with clinicopathological parameters of HCC, including tumor size, venous infiltration, TNM stage and Edmondson Steiner grading (*P*<0.05, respectively, Table 2). In addition, HCC patients with higher HDAC3 expression had worse overall survival rate (*P*<0.05, Figure 2C) and recurrence-free survival rate (*P*<0.05, Figure 2D), which were consistent with the data from Kaplan-Meier Plotter (*P*<0.05, Supplementary Figure 3B). Taken together, these data demonstrate that HDAC3 is upregulated in HCC, and highly expressed HDAC3 correlates with poor prognosis of HCC patients.

### HDAC3 is induced by hypoxia in a HIF-1-dependent manner in HCC cells

**Figure 3:**
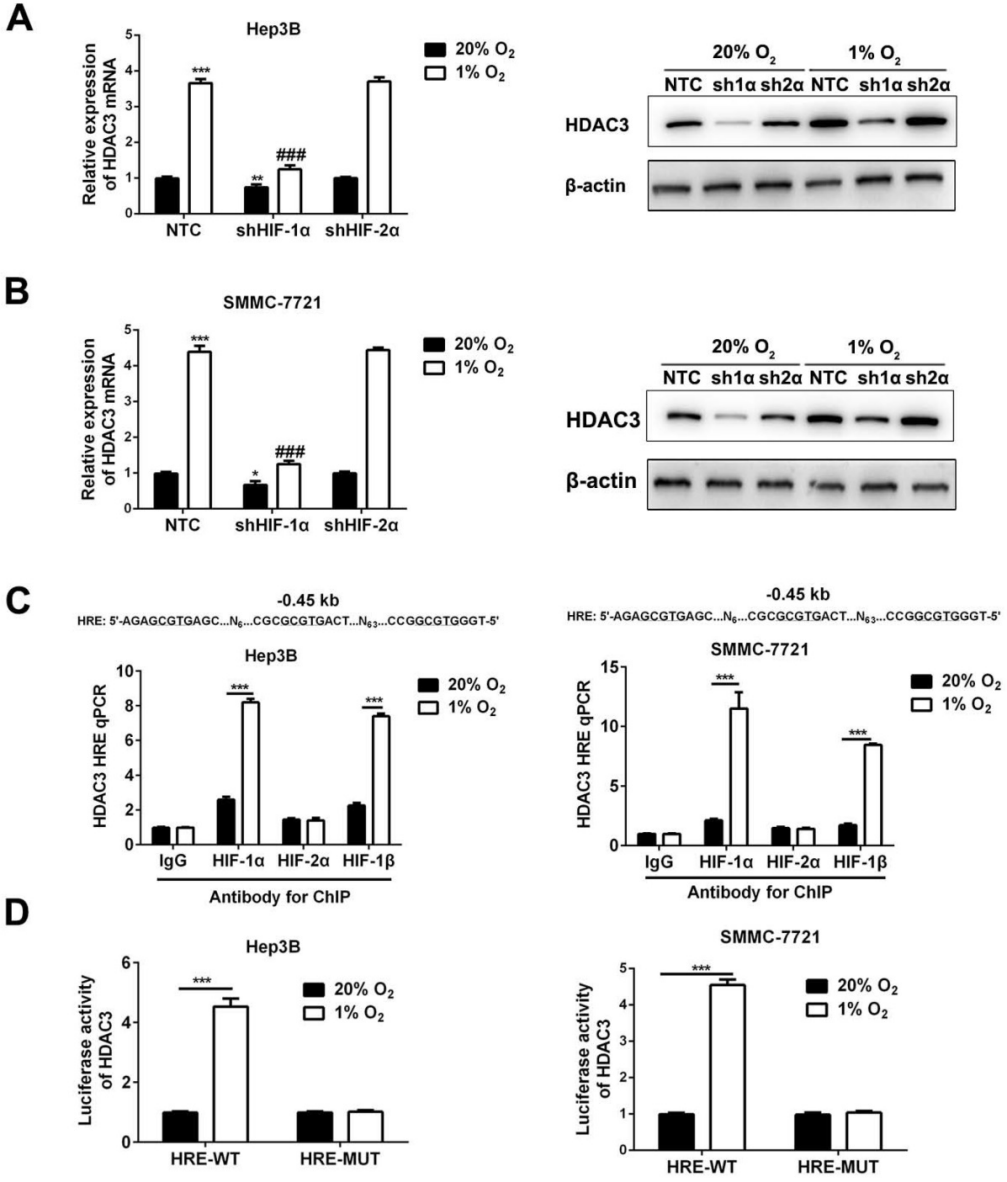
** HDAC3 is induced by hypoxia in a HIF-1-dependent manner in HCC cells.** (**A**) RT-qPCR analysis (left panel, mean ±SD; *n* = 3) and Western blot (right panel) were applied to test HDAC3 expression in the indicated subclones of Hep3B. **P < 0.01, ***P < 0.001 vs. NTC at 20% O_2_; ###*P* < 0.001 vs. NTC at 1% O_2_ (two-way ANOVA). (**B**) RT-qPCR analysis (left panel) and Western blot (right panel) were applied to test HDAC3 expression in the indicated subclones of SMMC7721. *P < 0.05, ***P < 0.001 vs. NTC at 20% O_2_; ###*P* < 0.001 vs. NTC at 1% O_2_ (two-way ANOVA). (**C**) Hep3B and SMMC-7721 cells were exposed to 20% or 1% O_2_ for 16h, and ChIP assays were performed using the indicated antibodies. Primers encompassing candidate HIF binding sites located 0.45 kb 5' to the HDAC3 gene transcription start site were used for qPCR and results were normalized to the first lane (mean ±SD; *n* = 3). ***P < 0.001, Student's *t* test. (**D**) The oligonucleotide spanning wild type or mutant type of HRE site was inserted into the reporter plasmid pGL2-promoter, in which a basal SV40 promoter drives firefly luciferase expression. Hep3B and SMMC-7721 cells were co-transfected with pGL2/HADC3-HRE and exposed to 20% or 1% O_2_ for 24h. The ratio of firefly/Renilla luciferase activity was analyzed (mean ± SD; *n* = 3). ***P < 0.001, Student's *t* test.

The HIF-1α-knockdown or HIF-2α-knockdown subclones of Hep3B and SMMC-7721 were exposed to 20% or 1% O_2_ for 24h and RNA was isolated for analysis by RT-qPCR. The result revealed that HDAC3 expression was induced by hypoxia, and the induction was abrogated by knockdown of HIF-1α, but not by knockdown of HIF-2α (*P*<0.05, Figure 3A). Consistently, Western blot of cells exposed to hypoxia for 48 hours showed decreased HDAC3 protein expression in the shHIF-1α subclone, but not in shHIF-2α subclone (*P*<0.05, Figure 3B). Furthermore, to determine whether HIFs directly bind to HDAC3 gene to activate its transcription, Hep3B or SMMC-7721 cells were exposed to 20% or 1% O_2_ for 16 hours, and ChIP assay indicated that both HIF-1α and HIF-1β were able to directly target the HRE site, while HIF-2α failed (*P*<0.05, Figure 3C). Next, to further verify the function of this HRE site, wild type or mutant type of pGL2/HDAC3-HRE was constructed, then co-transfected with pSV-Renilla into Hep3B and SMMC-7721 cells to conduct the luciferase activity assay. The ratio of firefly/Renilla luciferase activity showed the activity was significantly enhanced in HCC cells with wild type of pGL2/HDAC3-HRE, but not in the cells with mutant type of pGL2/HDAC3-HRE (*P*<0.05, Figure 3D). Taken together, these findings demonstrated that hypoxia induces HDAC3 expression that is directly regulated by HIF-1 in HCC cells.

### HDAC3 inhibits miR-627-5p expression by deacetylation in HCC

**Figure 4:**
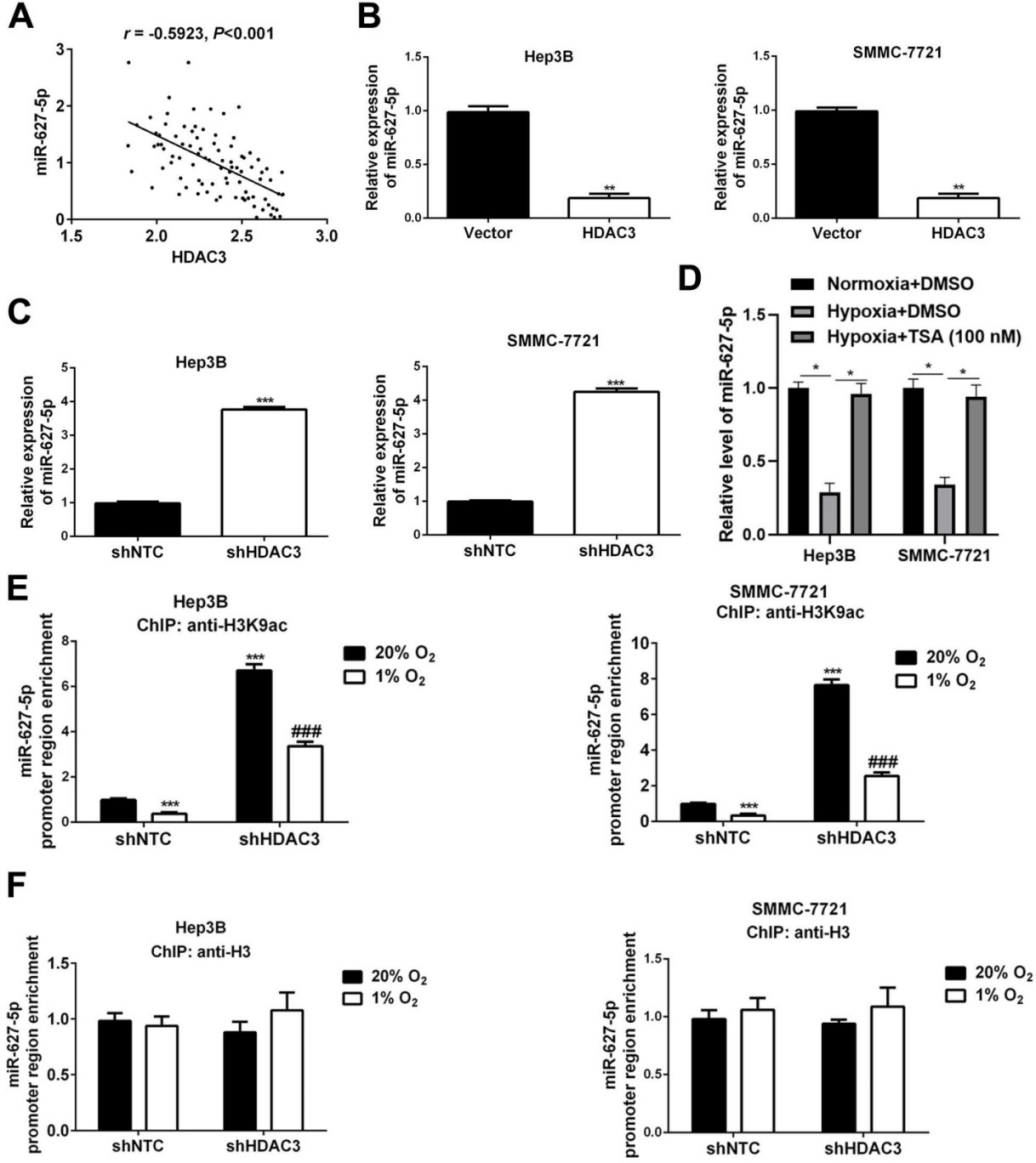
** HDAC3 inhibits miR-627-5p expression by deacetylation in HCC.** (**A**) Pearson correlation analysis was applied to investigate the correlation between HDAC3 expression and miR-627-5p expression in HCC tissues (*r*=-0.5923, *P*<0.001). (**B**) RT-qPCR analysis was applied to test the expression of miR-627-5p in Hep3B and SMMC-7721 with pcDNA/HDAC3 plasmid or the empty vector (mean ± SD; *n* = 3). ***P < 0.001, Student's *t* test. (**C**) RT-qPCR analysis was applied to test the expression of miR-627-5p in HDAC3 knockdown subclone of Hep3B and SMMC-7721 (mean ± SD; *n* = 3). ***P < 0.001, Student's *t* test. (**D**) Hep3B and SMMC7721 cells were exposed to normoxia or hypoxia, with the presence of DMSO or histone deacetylase inhibitor trichostatin A (TSA). And RNA was isolated for RT-qPCR analysis of miR-627-5p (mean ± SD; *n* = 3). *P < 0.05, two-way ANOVA. Hep3B and SMMC-7721 cells were exposed to 20% or 1% O_2_ for 16h, and ChIP assays were performed using antibodies against H3K9ac (**E**) and H3 (**F**). Primers encompassing MIR627 promoter region of the MIR627 gene was used for qPCR and results were normalized to the first lane (mean ±SD; *n* = 3). ***P < 0.001 vs. NTC at 20% O_2_; ###*P* < 0.001 vs. NTC at 1% O_2_, two-way ANOVA.

Next, we attempted to investigate whether miR-627-5p was regulated by HDAC3 in HCC cells. Pearson correlation analysis showed that there existed a negative correlation between HDAC3 expression and miR-627-5p in HCC tissues (*r*=-0.5923, *P*<0.001, Figure 4A). And, HDAC3 overexpressing and knockdown subclones in Hep3B and SMMC7721 were constructed, and the efficiencies were verified by RT-qPCR and Western blot (*P*<0.05, Supplementary Figure 4). Then, we found that overexpressed HDAC3 inhibited miR-627-5p expression in Hep3B and SMMC7721 cells (*P*<0.05, Figure 4B), while downregulated HDAC3 promoted miR-627-5p expression (*P*<0.05, Figure 4C). Furthermore, we determined that miR-627-5p expression was decreased by hypoxia in Hep3B and SMMC7721 cells, and the repression was blocked by the histone deacetylase inhibitor trichostatin A (TSA) (*P*<0.05, Figure 4D). ChIP assay by antibody against H3K9ac showed decreased histone H3 acetylation across the MIR627 promoter region under hypoxic conditions that was concomitant with MIR627 downregulation (*P*<0.05, Figure 4E). And the acetylation level and MIR627 expression were increased in HDAC3-knockdown subclones (*P*<0.05, Figure 4E). However, there was no any changes in total H3 acetylation levels under hypoxic conditions or in HDAC3 knockdown subclones (Figure 4F). ChIP assay revealed the interaction of HDAC3 and promoter region of miR-627-5p in HCC cells (*P*<0.05, Supplementary Figure 5). Taken together, these data demonstrate that miR-627-5p expression is inhibited by HDAC3-mediated deacetylation in HCC cells.

### Hypoxia inhibited miR-627-5p expression by HIF-1α/HDAC3/miR-627-5p pathway in HCC

**Figure 5:**
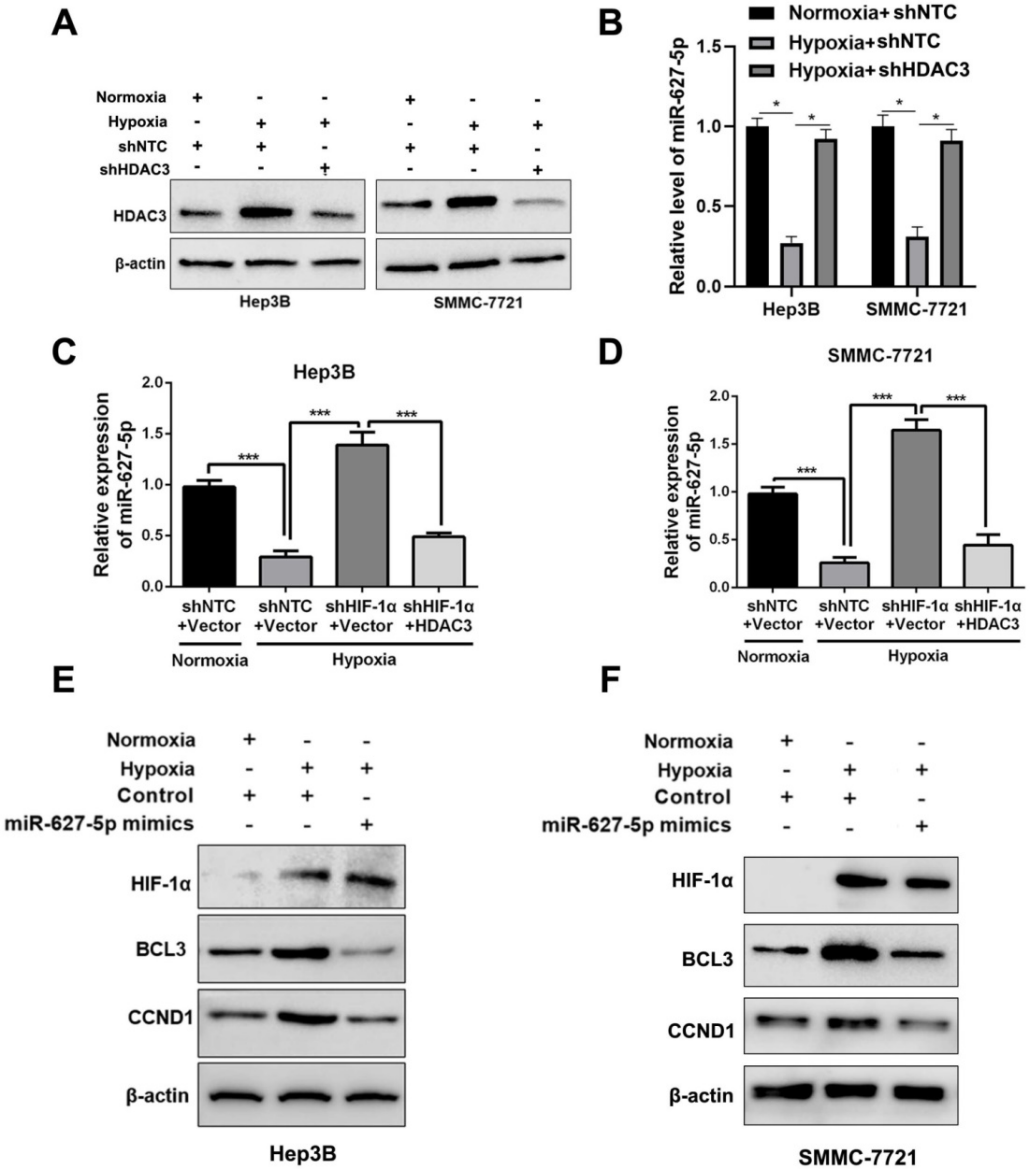
** Hypoxia inhibits miR-627-5p expression by HIF-1α/HDAC3/miR-627-5p pathway in HCC.** (**A**) HDAC3 knockdown or NTC subclones of Hep3B and SMMC-7721 were exposed to normoxia or hypoxia condition. And Western blot was applied to analyze the protein expression change of HDAC3. (**B**) RT-qPCR analysis was applied to test HDAC3 mRNA expression change in HDAC3 knockdown or NTC subclones of Hep3B and SMMC-7721 cells, which were exposed to normoxia or hypoxia (mean ±SD; *n* = 3). *P < 0.05, two-way ANOVA. Co-transfection of HIF-1α and pcDNA/HDAC3 plasmid or the corresponding controls was conducted in Hep3B (**C**) and SMMC-7721 (**D**) cells, and the subclones were exposed to normoxia or hypoxia. RT-qPCR analysis was applied to test miR-627-5p expression change (mean ±SD; *n* = 3). ***P < 0.001, two-way ANOVA. Hep3B (**E**) and SMMC-7721 (**F**) with miR-627-5p mimics or control were exposed to normoxia or hypoxia condition. And Western blot was applied to analyze the protein expression change of HIF-1α, BCL3 and CCND1.

Next, we attempted to explore whether hypoxia regulated miR-627-5p expression through HIF-1α/HDAC3/miR-627-5p pathway in HCC cells. As expected, the induction of HDAC3 expression by hypoxia was abrogated by HDAC3 shRNA (*P*<0.05, Figure 5A). Then, RT-qPCR analysis showed that miR-627-5p was suppressed by hypoxia, while the repression was reversed by HDAC3-knockdown (*P*<0.05, Figure 5B). Additionally, we found that HIF-1α-knockdown reversed the repression of miR-627-5p by hypoxia, and overexpressed HDAC3 blocked this reversion under hypoxia in Hep3B and SMMC-7721 cells (*P*<0.05, Figure 5C and 5D). Furthermore, BCL3 and CCND1, which were negatively regulated by miR-627-5p, were induced by hypoxia, and the inductions were abrogated by miR-627-5p mimics under hypoxia (Figure 5E and 5F). The IHC staining of HIF-1α, HDAC3, BCL3, and CCND1 and H&E staining was performed in the serial section of the HCC sample and we found the positive staining of HIF-1α, HDAC3, BCL3, and CCND1 in the same region (Supplementary Figure 6). Thus, we demonstrate that hypoxia inhibits miR-627-5p expression by HIF-1α/HDAC3/miR-627-5p pathway in HCC.

### miR-627-5p mediates hypoxia-induced HCC progression

**Figure 6:**
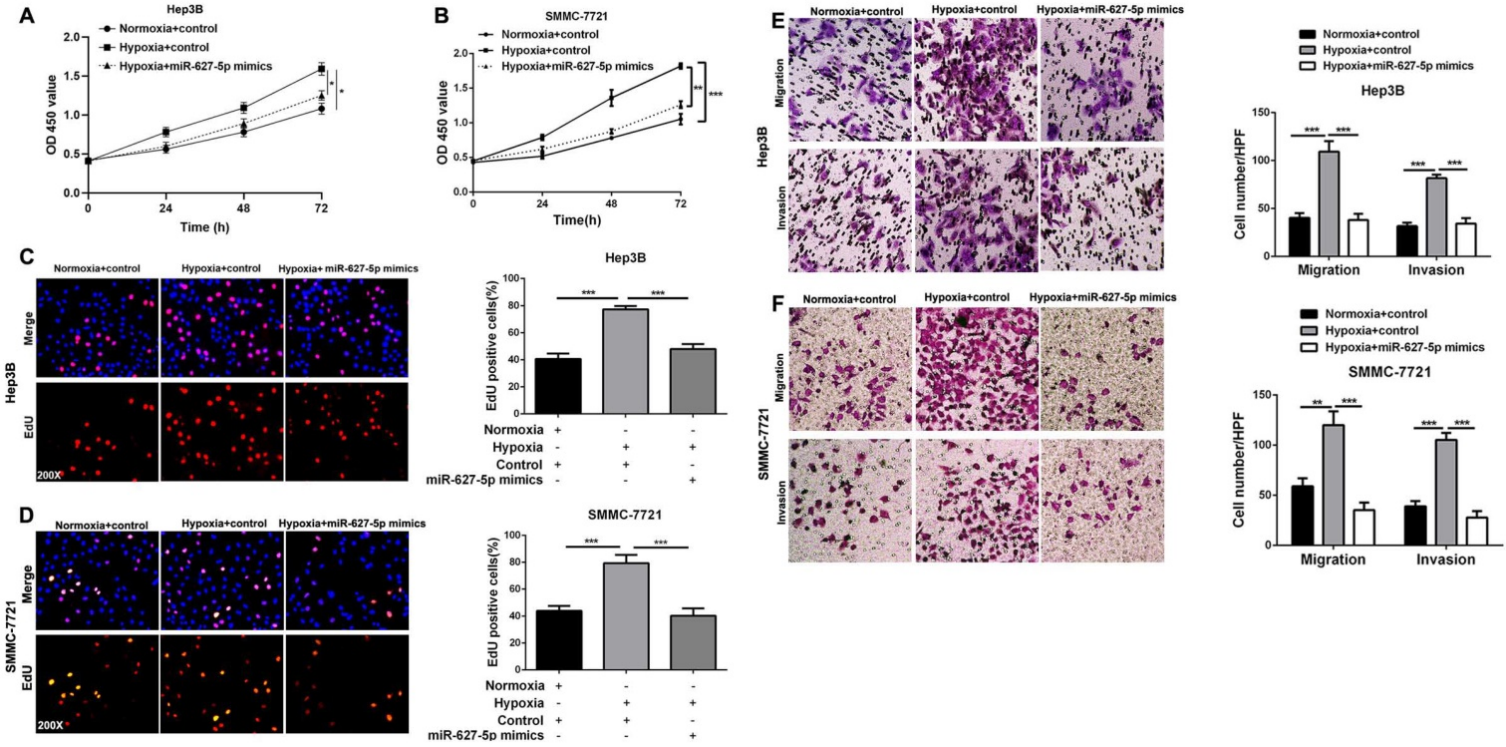
** miR-627-5p mediates hypoxia-induced HCC progression.** Hep3B and SMMC-7721 with miR-627-5p mimics or control were exposed to normoxia or hypoxia condition. (**A, B**) CCK-8 assay, (**C, D**) EdU cell proliferation assay and (**E, F**) Transwell migration and invasion assays were conducted to test the cells growth, migration and invasion (mean ±SD; *n* = 3). *P < 0.05, ***P < 0.001, two-way ANOVA.

It's well known that hypoxic condition promotes HCC cells growth, migration and invasion, and our previously published data demonstrated that miR-627-5p inhibits HCC progression 21, 24. Here, we attempted to explore whether miR-627-5p mediated the effects of hypoxia on HCC progression. CCK-8 assay showed that hypoxia promoted Hep3B and SMMC-7721 cells viability, and overexpressed miR-627-5p abrogated the promotion by hypoxia (*P*<0.05, Figure 6A and 6B). Consistently, EdU assay showed that hypoxia enhanced Hep3B and SMMC-7721 cells proliferation, and overexpressed miR-627-5p blocked the promotion by hypoxia (*P*<0.05, Figure 6C and 6D). In addition, Transwell migration and invasion assay showed that Hep3B and SMMC-7721 cells migration and invasion abilities were promoted by hypoxia, while the promotion was blocked by overexpressed miR-627-5p mimics (*P*<0.05, Figure 6E and 6F). Taken together, these findings demonstrate that miR-627-5p mediates hypoxia-induced HCC progression.

## Discussion

A growing number of studies have shown that miRNAs play critical roles in HCC tumorigenesis, which provides new insights into the gene target therapy for HCC 25. In our previous studies, miR-627-5p has been identified as a tumor suppressor in HCC, and we have also confirmed that BCL3 is the direct downstream target of miR-627-5p 21. In addition, BCL3/CCND1 pathway regulated by miR-627-5p participates HCC progression 21, 22. Intratumoral hypoxia is a powerful driving force for HCC progression 26. In response to hypoxia, hypoxia-inducible factors (HIFs) activate transcription of hundreds of genes that play key roles in angiogenesis, proliferation, invasion and metastasis 6, 13, 26. MiRNAs, a cluster of genes that have no capacity to encode protein, have also been reported to be regulated by hypoxia directly or indirectly 8, 9, 27. Thus, based on the findings from Chip Analysis on Gene Expression Profile that BCL3 and CCND1 were induced by hypoxia in Hep3B cells, we hypothesized that miR-627-5p was repressed by hypoxia. Then, the kinetic analysis of miR-627-5p expression in HCC cells exposed to hypoxia for different duration time revealed that miR-627-5p expression was repressed by hypoxia in a time-dependent manner. And miR-627-5p was negatively regulated by HIF-1α, rather than HIF-2α. Subsequently, we attempted to explore whether miR-627-5p was regulated by HIF-1α through the binding of HIF-1α and HRE sites. Unfortunately, both ChIP assay and luciferase reporter assay showed that HIF-1α was not able to promote MIR627 gene transcription directly through the HRE sites. Thus, we conclude that miR-627-5p is not a HIF-1α downstream direct target gene, and there could exist a mediator between HIF-1α and miR-627-5p.

Histone deacetylases (HDACs) have been identified as important epigenetic mediators in cancer cells 16. The acetylation of histones decreases the net positive charge of histones, leading to chromatin decondensation, which then promotes gene transcription 16. HDAC3 is a member of HDACs, and plays critical roles in cancer progression 19. In this study, we found that HDAC3 was frequently upregulated in HCC tissues both from our own cohort and the TCGA dataset. And HDAC3 expression was significantly related to tumor size, venous infiltration, TNM stage and Edmondson Steiner grading. In addition, HCC patients with higher HDAC3 expression had worse prognosis. Furthermore, our findings demonstrate that HDAC3 was a HIF-1α target gene in HCC cells. And hypoxia promoted HDAC3 expression through the binding of HIF-1 and HRE site.

It has been reported that HDAC3 could act as a mediator for gene expression regulation by hypoxia 28, 29. For example, hypoxia-driven histone deacetylase 3 (HDAC3), as an upstream regulatory mechanism, is critical for the downregulation of RUNX1-IT1 in HCC 28. HIF-1α-induced HDAC3 is essential for hypoxia-induced EMT and metastatic phenotypes 30. Thus, we attempted to explore the correlation between HDAC3 and miR-627-5p. Data showed that there existed a negative correlation between HDAC3 expression and miR-627-5p expression in HCC tissues. And miR-627-5p was negatively regulated by HDAC3 in HCC cells. Furthermore, we found that miR-627-5p was repressed by hypoxia, but the repression was blocked by the histone deacetylase inhibitor trichostatin A (TSA) or HDAC3 shRNA, suggesting that HDAC3-medated deacetylation mediates the repression of miR-627-5p by hypoxia. In addition, we also tested that whether HDAC3 knockdown affected deacetylation level of MIR627 gene promoter region. As expected, ChIP assay by antibody against H3K9ac showed decreased histone H3 acetylation across the MIR627 promoter region under hypoxic conditions, and the repression was reversed by HDAC3 shRNA. Thus, these findings demonstrate that hypoxia repressed miR-627-5p expression through HDAC3-medated deacetylation in HCC cells. Then, the further rescue experiments determined the HIF-1α/HDAC3/miR-627-3p/BCL3/CCND1 pathway in HCC cells. And miR-627-5p mediated the promotion effects of hypoxia on HCC cells.

In conclusion, this study revealed that miR-627-5p is repressed by hypoxia, and HDAC3 is induced by hypoxia. And Hypoxia regulated miR-627-5p through HIF-1α/HDAC3/miR-627-5p pathway, during which HDAC3-deacetylation plays a critical role. Finally, we determined a HIF-1α/HDAC3/miR-627-3p/BCL3/CCND1 pathway in HCC cells. The current study suggests a novel therapeutic strategy for HCC patients.

## Supplementary Material

Supplementary figures.

## Figures and Tables

**Table 1:** Part of the hypoxia-induced genes in Hep3B cells

Gene	*P*-value	FDR	Fold change	Regulation
BNIP3	0.000156959	0.005707546	8.8845108	Up
GLUT1	0.001625273	0.015806138	2.859151	Up
CA9	0.000842376	0.011312339	2.4555879	Up
PDK1	0.000430161	0.008177802	6.0179259	Up
LOXL2	0.000463037	0.008471111	3.8281676	Up
ANGPTL4	0.002251054	0.019053964	6.1695497	Up
PGK1	0.005085153	0.030014764	3.6155171	Up
PLOD1	0.000632947	0.009801999	3.5783099	Up
P4HA1	0.00082263	0.011230358	6.5723144	Up
P4HA2	0.001006396	0.012454973	3.7229072	Up
BCL3	0.00704518	0.036759569	2.5207837	Up
CCND1	0.000107998	0.005079804	3.773573	Up

**Table 2:** Clinicopathological correlation of HDAC3 expression in the HCC cases

Clinicopathologic features	*n*	HDAC3 expression		*P*
		High (*n*=45)	Low (*n*=45)	
**Age (year)**				0.667
≤50	36	19	17	
>50	54	26	28	
**Sex**				0.334
Male	79	41	38	
Female	11	4	7	
**HBV infection**				0.134
No	13	4	9	
Yes	77	41	36	
**Serum AFP level (ng/ml)**				0.107
≤20	27	10	17	
>20	63	35	28	
**Tumor size (cm)**				0.020*
≤5	41	15	26	
>5	49	30	19	
**No. of tumor nodules**				0.098
1	74	34	40	
≥2	16	11	5	
**Cirrhosis**				0.081
Absent	21	14	7	
Present	69	31	38	
**Venous infiltration**				0.006*
Absent	43	15	28	
Present	47	30	17	
**Edmondson-Steiner grading**				0.014*
I+II	68	29	39	
III+IV	22	16	6	
**TNM stage**				0.030*
I+II	67	29	38	
III+IV	23	16	7	

*P<0.05.
