# Supplementary material for: Repression of the miR-627-5p by histone deacetylase 3 contributes to hypoxia-induced hepatocellular carcinoma progression
Source: J Cancer. 2021 Jul 3;12(17):5320–30. doi: 10.7150/jca.58697 (PMC8317525; doi:10.7150/jca.58697)
Supplement: Supplementary file 1 — Supplementary figures. [file jcav12p5320s1.pdf]

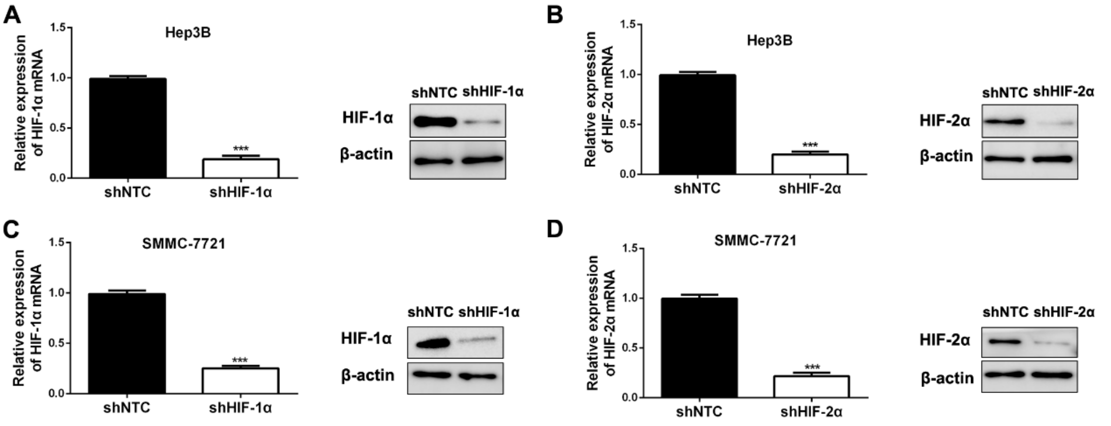

Supplementary Figure 1 HIF-1α/2α knockdown subclones are established. (A, B) Hep3B cells were stably transfected with lentiviral vectors encoding a non-targeting control (NTC) short hairpin RNA (shRNA) or shRNA targeting either HIF-1α (shHIF-1α) or HIF-2α (shHIF-2α). RT-qPCR and Western blot were applied to verify the knockdown efficiency. (C, D) SMMC-7721 cells were stably transfected with lentiviral vectors encoding a non-targeting control (NTC) short hairpin RNA (shRNA) or shRNA targeting either HIF-1α (shHIF-1α) or HIF-2α (shHIF-2α). RT-qPCR and Western blot were applied to verify the knockdown efficiency. (mean ± SD; n = 3). ***$P < 0.001$, Student's t test.

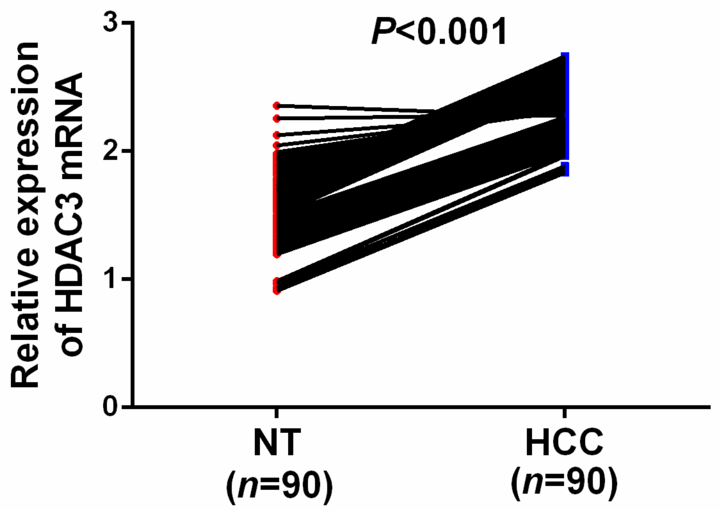

Supplementary Figure 2 The expression of HDAC3 mRNA in 90 pairs of HCC and non-tumor tissues.

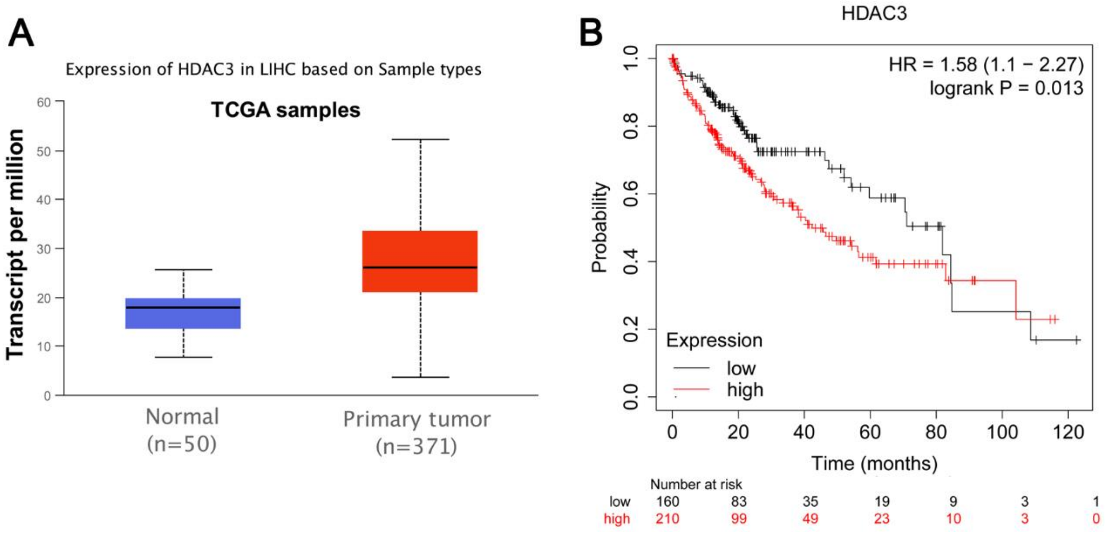

Supplementary Figure 3 The expression and prognostic significance of HDAC3 in HCC based on TCGA data. (A) TCGA data from GEPIA platform showed that HDAC3 expression in HCC tissues (*n*=371) was significantly higher than that in normal tissues (*n*=50). (Student's *t* test). (B) Data from Kaplan-Meier Plotter showed that HCC patients with higher HDAC3 expression had worse survival rate (Log-rank test).

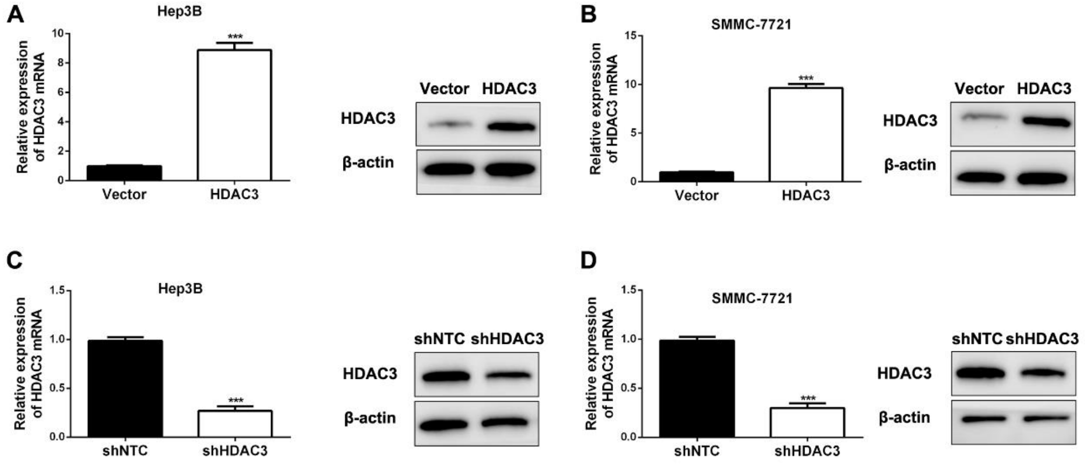

Supplementary Figure 4 HDAC3 knockdown or overexpressing subclones are established. The pcDNA/HDAC3 plasmid was used to overexpress HDAC3 expression of Hep3B cells (A) and SMMC-7721 (B) cells, and empty vector was the control. RT-qPCR and Western blot were applied to verify the knockdown efficiency. (mean ± SD; n = 3). The lentiviral vectors encoding a NTC shRNA (shNTC) or shRNA targeting HDAC3 (shHDAC3) was applied to establish the HDAC3 knockdown or control subclones in Hep3B (C) and (D) SMMC-7721 cells. RT-qPCR and Western blot were applied to verify the knockdown efficiency. (mean ± SD; n = 3). $^{***}P < 0.001$,

Student's *t* test.

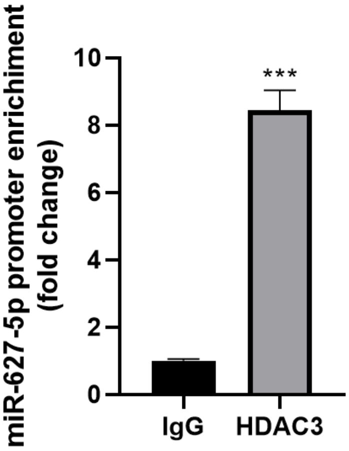

Supplementary Figure 5 The interaction of HDAC3 and promoter region of miR-627-5p in Hep3B cells as suggested by ChIP. ***$P < 0.001$, Student's *t* test.

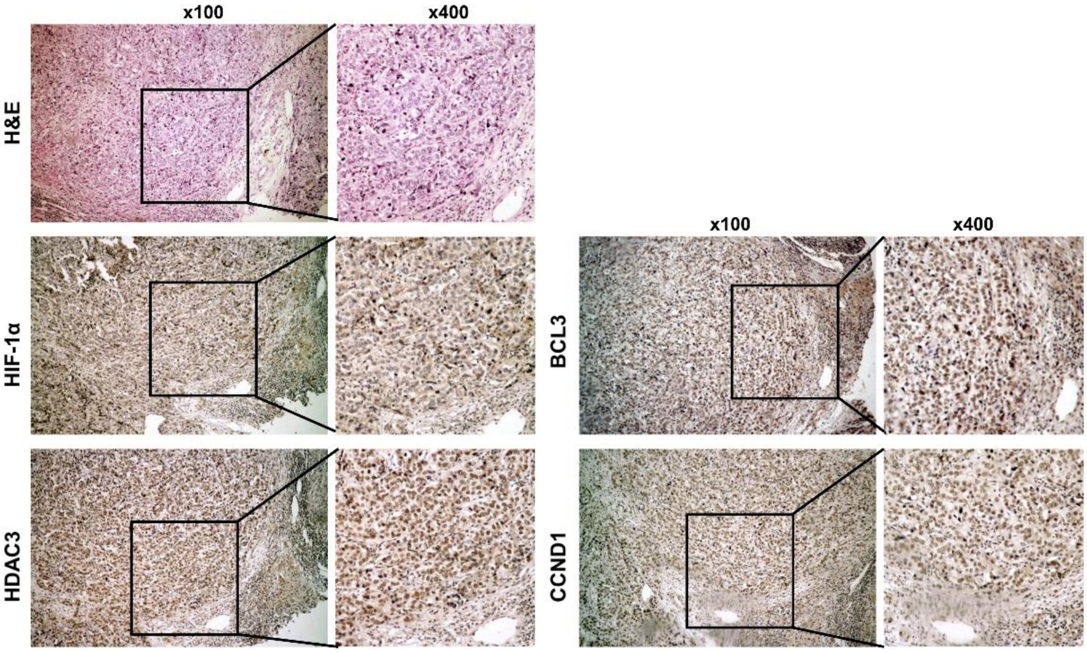

Supplementary Figure 6 The IHC staining of HIF-1α, HDAC3, BCL3, and CCND1 and H&E staining is performed in the serial section of the HCC sample.
